# Identification of Promotor and Exonic Variations, and Functional Characterization of a Splice Site Mutation in Indian Patients with Unconjugated Hyperbilirubinemia

**DOI:** 10.1371/journal.pone.0145967

**Published:** 2015-12-30

**Authors:** Neha Gupta, Mercilena Benjamin, Anjana Kar, Sachin Dev Munjal, Aditya N. Sarangi, Ashwin Dalal, Rakesh Aggarwal

**Affiliations:** 1 Department of Gastroenterology, Sanjay Gandhi Postgraduate Institute of Medical Sciences, Lucknow, 226014, India; 2 Biomedical Informatics Center, Sanjay Gandhi Postgraduate Institute of Medical Sciences, Lucknow, 226014, India; 3 Diagnostics Division, Centre for DNA Fingerprinting and Diagnostics, Hyderabad, 500001, India; 4 Graduate Studies, Manipal University, Manipal, 576104, Karnataka, India; Cancer Research Centre of Lyon, FRANCE

## Abstract

**Background:**

Mild unconjugated hyperbilirubinemia (UH), due to reduced activity of the enzyme uridine diphosphoglucuronate-glucuronosyltransferase family, polypeptide 1 (UGT1A1), is a common clinical condition. Most cases are caused by presence in homozygous form of an A(TA)_7_TAA nucleotide sequence instead of the usual A(TA)_6_TAA sequence in promoter region of the *UGT1A1* gene. In some cases, other genetic variations have been identified which differ between populations. There is need for more data on such genetic variations from India.

**Methods:**

DNA from subjects with unexplained persistent or recurrent UH was tested for the presence of TA promoter insertions. In addition, all five exons and splicing site regions of *UGT1A1* gene were sequenced. Several bioinformatics tools were used to determine the biological significance of the observed genetic changes. Functional analysis was done to look for effect of a splice site mutation in *UGT1A1*.

**Results:**

Of 71 subjects with UH (68 male; median age [range], 26 [16–63] years; serum bilirubin 56 [26–219] μM/L, predominantly unconjugated) studied, 65 (91.5%) subjects were homozygous for A(TA)_7_TAA allele, five (7.0%) were heterozygous, and one (1.4%) lacked this change. Fifteen subjects with UH had missense exonic single nucleotide changes (14 heterozygous, 1 homozygous), including one subject with a novel nucleotide change (p.Thr205Asn). Bioinformatics tools predicted some of these variations (p.Arg108Cys, p.Ile159Thr and p.Glu463Val) to be deleterious. Functional characterization of an exonic variation (c.1084G>A) located at a splice site revealed that it results in frameshift deletion of 31 nucleotides and premature truncation of the protein.

**Conclusion:**

Our study revealed several single nucleotide variations in *UGT1A1* gene in Indian subjects with UH. Functional characterization of a splice site variation indicated that it leads to disordered splicing. These variations may explain UH in subjects who lacked homozygous A(TA)_7_TAA promoter alleles.

## Introduction

Gilbert syndrome (GS) and Crigler-Najjar syndrome 2 (CNS2) are both characterised by occurrence of mild to moderate unconjugated hyperbilirubinemia (UH) in the absence of hemolysis and liver disease, with bilirubin levels being higher in the latter. In persons with GS, the elevated bilirubin levels may persist for several years, or may occur only intermittently [1]. These conditions are associated with reduced activity of the enzyme uridine diphosphoglucuronate-glucuronosyltransferase family, polypeptide 1 (UGT1A1) which converts hydrophobic unconjugated bilirubin into its mono- and di-glucuronides; the latter are hydrophilic and can be easily eliminated from the body in bile and to some extent in urine.

UH is a benign condition, without any major adverse clinical consequences. In fact, many population-based studies have shown that individuals with GS have a reduced risk of cardiovascular disease [2]. However, the UGT1A1 enzyme also modulates metabolism of some exogenous (drugs) and endogenous substrates. Thus, a reduced activity of this enzyme predisposes individuals with UH to toxicity following the administration of some drugs such as irinotecan [3], which is used for the treatment of cancers, in particular colon cancer. This has led to a renewed interest in UH in recent years.

Most persons with UH, particularly those with GS, show a homozygous polymorphism in promoter region of the *UGT1A1* gene, wherein a dinucleotide (TA) is inserted in the TATA box-like sequence, leading to an A(TA)_7_TAA nucleotide sequence instead of the more frequent A(TA)_6_TAA sequence [4]. This insertion polymorphism, also known as UGT1A1*28, is associated with decreased activity of UGT1A1 enzyme. However, several persons with this variant allele in homozygous state do not have elevated bilirubin levels, indicating that mere presence of the variant promoter region may not be adequate for appearance of UH phenotype [4].

Some individuals with UH either entirely lack the variant A(TA)_7_TAA allele or have only one such allele. In such individuals, several other variations in the promoter and coding regions of the *UGT1A1* gene have been identified [5]. Such variations vary widely between populations.

Data on variations of the *UGT1A1* gene in Indian population are fairly limited [6, 7]. We therefore undertook a study of genetic variations in this gene in a northern Indian group of subjects with UH.

## Methods

### Subjects

The study enrolled subjects with UH, which was diagnosed based on the presence of persistent or recurrent increase in serum bilirubin (total serum bilirubin ≥26 μM/L) which was predominantly unconjugated, in the absence of other symptoms of hepatobiliary disease, absence of clinical and laboratory features of hemolysis, and with normal findings at ultrasonography of the liver and biliary tree and other liver function tests. The study was approved by Ethics Committee of the Sanjay Gandhi Postgraduate Institute of Medical Sciences, Lucknow, India and each subject provided a written informed consent; for one subject aged below 18 years of age (the legal age of consent in India), a parent provided written consent and the subject himself provided a written assent.

From each subject, venous blood was collected in plain and EDTA vials. Serum total and direct bilirubin levels, and alanine aminotransferase activity were measured using commercial test kits (Randox Laboratories, Crumlin, UK). In addition, genomic DNA was extracted from EDTA blood using ethanol precipitation method.

### Genetic testing

DNA from all the patients was analyzed for the presence of TA insertion in the TATA box region of the *UGT1A1* gene. For this, a DNA fragment encompassing the TATA box region was amplified using a fluorochrome (6-FAM)-labelled forward primer and an unlabelled reverse primer (Table 1) [8, 9, 10]. The amplification products were then analyzed on ABI Prism 3130 Genetic Analyzer (Applied Biosystems, Foster City, CA, USA) along with a molecular weight marker (LIZ 120 Size Standard; Applied Biosystems) using GeneMapper software v4.1 (Applied Biosystems). The PCR product was expected to be 90 base-pair (bp) and 92 bp long, for alleles with six and seven TA repeats, respectively.

**Table 1:** Primers for PCR amplification of various segments of the *UGT1A1* gene.

*Gene region amplified*	*Sequence (5’ to 3’)*	*PCR product size (bp)*	*Reference*
**Promoter region (for fragment length analysis)**	6-FAM—TAA CTT GGT GTA TCG ATT GGT TTT TG	90	[9]
	ACA GCC ATG GCG CCT TTG CT		
**Promoter region (for sequencing)**	AAG TGA ACT CCC TGC TAC CTT	253	[10]
	CCA CTG GGA TCA ACA GTA TCT		
**Regulatory region**	CTA GCC ATT CTG GAT CCC TTG	367	[9]
	TTT TGA GAT CTG AGT TCT CTT CAC CTC		
Exon 1*	TAT AAG TAG GAG AGG GCG AAC C	588	[9]
	TCA AAT TCC AGG CTG CAT G		
	GGC CTC CCT GGC AGA AAG	617	[9]
	ATG CCA AAG ACA GAC TCA AAC C		
**Exon 2**	TCT ATC TCA AAC ACG CAT GCC	374	[9]
	GGC AGG GAA AAG CCA AAT CTA		
**Exon 3**	TTG CCA GTC CTC AGA AGC CTT	423	[9]
	ATG CCC TTG CAG AAA TTT GC		
**Exon 4**	TGC AAG GGC ATG TGA GTA ACA	553	[9]
	AAG CCA AGA TTG CAC CAC TG		
**Exon 5**	GAG GAT TGT TCA TAC CAC AGG	436	[8]
	TGA ATT TAA CAC TGA TTC TGT T		
**Primers for generating pCAS2 mini-gene constructs**			
**UGT1A1-Ex3-*Bam*HIF**	GCATGCGGATCCCGGAAGTTGCCAGTCCTCAG	475	
**UGT1A1-Ex3-*Mlu*IR**	CTAAGTACGCGTGTGTTACTCACATGCCCTTGC		
**Primers for pCAS2 RT-PCR analysis**			
**pCAS_P1_F**	CTCCGCAGGTCCGCT		[11]
**pCAS_P2_R**	ATTGGTTGTTGAGTTGGTTGTC		

6-FAM = 6-carboxyfluorescein

*Exon 1, being long, was amplified as two overlapping amplicons; the sequences obtained from these were then merged

To confirm the results of this assay, a larger DNA segment surrounding the TATA box was amplified using another set of primers from DNA of one subject each with homozygous state for the wild-type and variant promoter allele (Table 1; expected PCR product lengths of 253 and 255 bp for A(TA)_6_TAA and A(TA)_7_TAA alleles, respectively). The amplicons were then sequenced in both directions, using the procedure described below.

In addition, genomic regions encompassing each of the five exons of the *UGT1A1* gene and the surrounding splice regions were amplified using polymerase chain reactions with primers that have been previously described (Table 1) [9,10]. The amplification products were purified using a commercial PCR purification kit (Invitrogen, Carlsbad, CA, USA) to remove the unbound primers and primer dimers. The purified products were subjected to DNA sequencing in both directions using the Big dye terminator v3.1 kit (Applied Biosystems, Carlsbad, CA, USA), and automated capillary electrophoresis using ABI Prism 3130 Genetic Analyzer.

Sequences obtained for each exon in the two directions were manually verified, and aligned with each other and with the reference genomic sequence for the *UGT1A1* gene **(**NG_002601.2). The data obtained were further analyzed using the GeneMapper, Seqscape (Life Technologies) and Finch TV (Geospiza; http://www.geospiza.com/Products/finchtv.shtml) softwares.

### Bioinformatics analysis

Each variation was looked up sequentially in the Single Nucleotide Polymorphism Database (dbSNP; www.ncbi.nlm.nih.gov/SNP) Human Genome Mutation Database (HGMD; http://www.hgmd.cf.ac.uk/), Exome Aggregation Consortium (ExAC; exac.broadinstitute.org) and ClinVar (http://www.ncbi.nlm.nih.gov/clinvar). Biological significance of genetic variations observed in the exonic regions was determined using seven tools, namely PROVEAN [12], SIFT [13], MutationAccessor [14], PolyPhen 2 [15], PhD-SNP [16], SNAP [17] and MutationTaster [18]. The effect of an observed nucleotide change which was located at the splice site was assessed using two tools, namely Human Splice Finder [19] and MutPred Splice [20].

### In-vitro analysis of mutation at splice site

**Fig 1:**
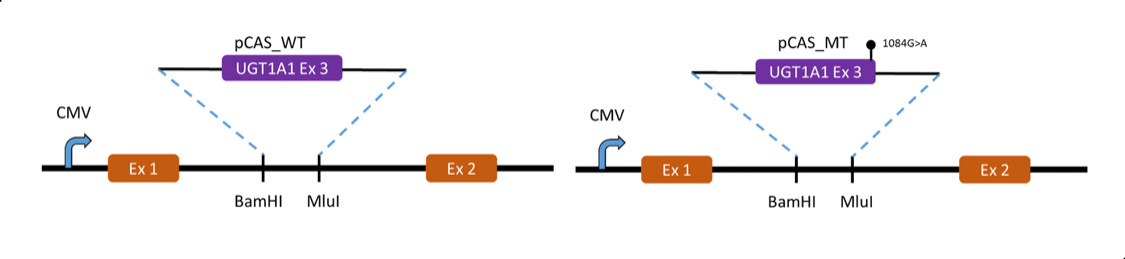
Molecular characterization of c.1084G>A mutation. Strategy for cloning UGT1A1 exon 3 including flanking intron sequence in BamHI and MluI restriction site at intron of pCAS minigene system.

An observed mutation c.1084G>A was evaluated for splicing defect using minigene construct pCAS2 [11, 21]. *UGT1A1* exon 3 including 162 bp and 190 bp of the preceding 5’- and the succeeding 3’-intronic regions, respectively, was PCR amplified from wild-type and patient DNA. The 5’-end of forward and reverse primers were designed to contain restriction sites for *Bam*HI and *Mlu*I to enable cloning of the PCR fragments into a pCAS2 vector. Cloning in pCAS2 minigene vector was done as per standard procedures to generate pCAS_WT and pCAS_MT, respectively (Fig 1). The two recombinant constructs as well as pCAS (as control) were separately transfected into COS7 cells using Lipofectamine 2000, transfecting reagent (Invitrogen, Carlsbad, CA) according to the manufacturer’s instructions. Cells were collected 18 h post-transfection and total RNA was extracted from each transfectant using RNeasy Mini Kit (Qiagen, Hilden, Germany), as per the manufacturer’s instructions. It was then subjected to RT-PCR using SuperScript III (Invitrogen, Carlsbad, CA) and evaluated on 2% agarose gel. Appropriate bands were purified from agarose gel using QIAquick Gel Extraction Kit (Qiagen, Hilden, Germany) and subjected to DNA sequencing on a 3130 genetic analyser (ABI, Foster City, CA) as per standard protocol. All primer sequences are listed in Table 1.

## Results

### Study subjects

The study included 71 unrelated subjects (68 male, 3 female) with UH. Their ages ranged from 16 to 63 (median 26) years, and serum bilirubin at presentation ranged between 26 and 219 (median 56) μM/L; in all the subjects, unconjugated bilirubin formed a predominant component of the total serum bilirubin.

### Promoter region polymorphism

**Fig 2:**
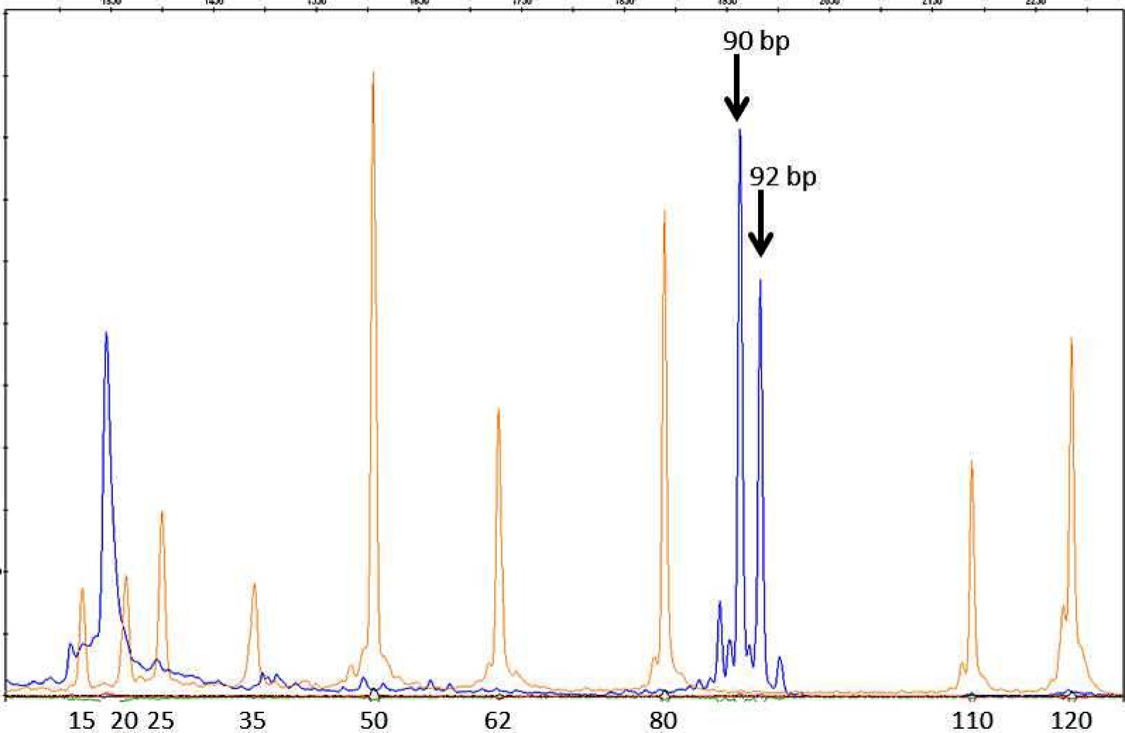
Results of fragment length analysis for detection of TA insertion in the TATA box region of the *UGT1A1* gene in a subject with heterozygous genotype. The blue line relates to fluorescence of 6-FAM; the two peaks marked with solid arrows indicate 90 base-pair and 92 base-pair products for A(TA)_6_TAA and A(TA)_7_TAA promoter alleles, respectively. The red line relates to fluorescent dye bound to the molecular weight markers, lengths for which are marked at the bottom.

The fragment length analysis method showed a clear distinction between the 90-bp and 92-bp peaks expected in subjects with A(TA)_6_TAA and A(TA)_7_TAA forms of the promoter gene (Fig 2). Sequencing of a larger region encompassing the TATA box confirmed the results of fragment analysis in one subject each with only 90-bp and 92-bp peaks.

Of the 71 subjects with UH, 65 (91.5%) were homozygous for A(TA)_7_TAA allele, five (7.0%) were heterozygous for this allele and one (1.4%) lacked this allele altogether (Table 2). The other known variants of the *UGT1A1* promoter region, i.e. A(TA)_5_TAA and A(TA)_8_TAA, were not identified in any subject.

**Table 2:** Details of promoter, exonic and splice site genetic variations in UGT1A1 gene identified in 71 Indian patients with unconjugated hyperbilirubinemia.

Promoter genotype	Exon	Nucleotide change	dbSNP / ExAC / ClinVar or HGMD ID	Amino acid change	Number of subjects	Total serum bilirubin (μM/L) †
**TA**_**7**_ **/TA**_**7**_ **(n = 65)**	No exonic variation				54	55 (26–106)
	1	c.211G>A	dbSNP: rs4148323; previously known to be associated with hyperbilirubinemia	p.Gly71Arg	5	68 (44–103)
	1	c.322C>T	dbSNP: rs587784538; ExAC: 2–234669255 C/T; ClinVar: RCV000147901.1	p.Arg108Cys	1	51
	1	c.476T>C	dbSNP: rs587784539 ; ExAC: 2–234669409 T/C; ClinVar: RCV000147902.1	p.Ile159Thr	1	89
	1	c.614C>A	Not previously described	p.Thr205Asn	1	41
	1	c.674T>G	dbSNP: rs35003977; pathogenic status unknown	p.Val225Gly	2	34–58
	5	c.1388A>Tǂ	dbSNP: rs72551358; ExAC: 2:234680991 A/T	p.Glu463Val	1	80
**TA**_**7**_ **/TA**_**6**_ **(n = 5)**	No exonic variation				2	60–70
	1	c.322 C>T	dbSNP: rs587784538; ExAC: 2–234669255 C/T; ClinVar: RCV000147901.1;	p.Arg108Cys	1	219
	3	c.1084 G>A (last base of the exon)	dbSNP: rs755218546; ExAC: 2:234676582 G/A; HGMD: CM098937	p.Gly362Ser	1	32
	5	c.1511 T>A	dbSNP: rs767732319; ExAC: 2:234681114 T/A	p.Phe504Tyr	1	67
**TA**_**6**_ **/TA**_**6**_ **(n = 1)**	3	c.1084 G>A* (last base of the exon)	dbSNP: rs755218546; ExAC: 2:234676582 G/A; HGMD: CM098937	p.Gly362Ser	1	132

All nucleotide positions are with respect to coding sequence with the first base of ATG numbered as 1.

*This one subject had a homozygous change; all other changes were heterozygous.

†Bilirubin levels are shown as median (range)

dbSNP, HGMD, ExAC and ClinVar IDs in the table refer to identification numbers in these databases, respectively.

ǂc.1388A>T was previously reported by Skierka et al (2013) [22]

### Exonic regions

Sequencing of all the five exons and the adjoining intronic regions of the *UGT1A1* gene revealed nucleotide changes in 15 of the 71 subjects with UH; details of these nucleotide changes and the consequent amino acid alterations in the UGT1A1 gene product are shown in Table 2. These genetic variations were present in heterozygous form in 14 patients and in homozygous state in one patient. All the nucleotide sequence changes were missense exonic variations, i.e. these were predicted to result in amino acid changes UGT1A1 gene product; one of these was located in the exonic part of the splice site. No changes were found in the intronic parts of the splice junctions.

Fourteen of these subjects had one of the polymorphisms that have been previously described (Table 2); the remaining one subject had a novel genetic variation (c.614C>A; p.Thr205Asn).

### Relationship of exonic changes with promoter site polymorphism

**Fig 3:**
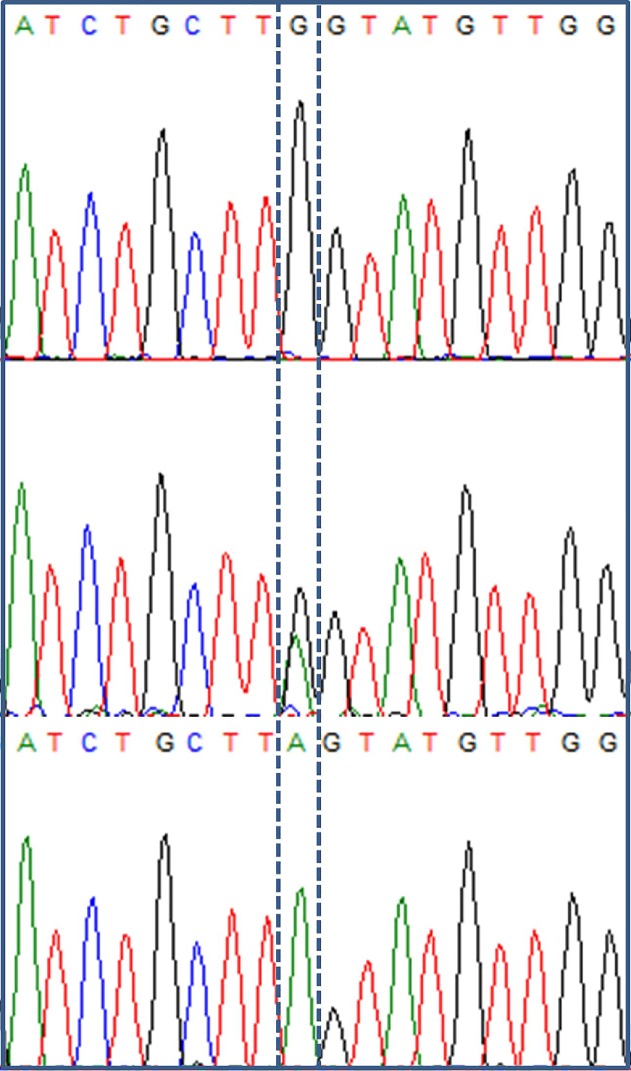
Electropherogram showing G/A mutation at nucleotide 1084 (located on the last nucleotide of exon 3). The nucleotide of interest is marked by a dotted box. The upper panel shows data from a subject homozygous for wild-type G nucleotide, the middle panel shows a subject with unconjugated hyperbilirubinemia who had heterozygous GA genotype, and the lower panel shows a subject with unconjugated hyperbilirubinemia who had homozygous mutant A genotype.

The only subject with UH who was homozygous for the wild-type A(TA)_6_TAA promoter allele (a 52-year-old woman, serum bilirubin = 132 μM/L) was also homozygous for a c.1084G>A (p.Gly362Ser) mutation (Fig 3). This mutation was located on the last nucleotide of the exon 3.

Of the five subjects with UH who had heterozygous A(TA)_7_TAA allele, three had exonic changes, namely c.322C>T (p.Arg108Cys; a 63-year-old man, serum bilirubin = 219 μM/L), c.1084G>A (p.Gly362Ser; 25-year-old man, 32 μM/L) and c.1511T>A (p.Phe504Tyr; 19-year-old man, 67 μM/L); the remaining two subjects did not have any detectable exonic variation.

Of the 65 subjects who were homozygous for A(TA)_7_TAA allele, 11 also had missense genetic changes, including p.Gly71Arg in five, p.Val225Gly in two, and p.Arg108Cys, p.Ile159Thr, p.Thr205Asn and p.Glu463Val in one each. Mean (±SD) serum total bilirubin level in these 11 patients (63±22 μM/L) was similar to that in the remaining 54 patients without additional exonic variations (56±17 μM/L).

### Bioinformatic analysis of exonic and c.1084G>A splice site genetic changes

The results of bioinformatics analysis for prediction of biological effect of various genetic changes observed in our study subjects are shown in Table 3. Of the seven bioinformatics tools used, all seven indicated that p.Glu463Val variation is likely to be deleterious, six indicated that p.Gly362Ser is likely to be deleterious, five indicated that p.Arg108Cys and p.Ile159Thr are likely to be deleterious, and three indicated that p.Gly71Arg and p.Phe504Tyr variations are likely to be deleterious; none of the web servers predicted p.Thr205Asn to be deleterious.

**Table 3:** Results of bioinformatics analysis of exonic variations.

Variation	ExAC variant frequency in South Asian population	Aminoacid Conservation scores		PROVEAN	SIFT	Mutation Assessor	Polyphen 2	PhD-SNP	SNAP	Mutation Taster	Number of methods showing deleterious effect
Transcript /protein		PhyloP	Phast-Cons	Prediction	Prediction	Prediction	Prediction	Prediction	Prediction	Prediction	
**c.211G>A (p.Gly71Arg)**	0.01902	2.13	0.01	Neutral	Tolerated	Low	Probably damaging	Disease	Non-neutral	Polymorphism	3/7
**c.322C>T (p.Arg108Cys)**	0.00036	0.17	0	Deleterious	Tolerated	Medium	Probably damaging	Disease	Neutral	Polymorphism	5/7
**c.476T>C (p.Ile159Thr)**	0.00067	5.3	0.99	Deleterious	Damaging	Medium	Possibly damaging	Neutral	Neutral	Disease causing	5/7
**c.614C>A (p.Thr205Asn)**	Novel variant	0.002	0.39	Neutral	Tolerated	Low	Benign	Neutral	Neutral	Polymorphism	0/7
**c.674T>G (p.Val225Gly)**	0.00212	0.63	0	Tolerated	Damaging	Neutral	Benign	Disease	Neutral	Polymorphism	2/7
**c.1084G>A (p.Gly362Ser)**	0.00036	6.12	1	Neutral	Damaging	Medium	Probably damaging	Disease	Non-neutral	Disease causing	6/7
**c.1388A>T (p.Glu463Val)**	0.00012	5.17	1	Deleterious	Damaging	High	Probably damaging	Disease	Non-neutral	Disease causing	7/7
**c.1511T>A (p.Phe504Tyr)**	0.00012	2.45	0.89	Neutral	Damaging	Low	Probably damaging	Neutral	Neutral	Disease causing	3/7

PROVEAN: PROtein Variation Effect Analyzer; SIFT: Sorting Intolerant from Tolerant; Polyphen 2: Polymorphism phenotyping2; PhD-SNP(Predictor of human Deleterious SNPs; SNAP: Screening for non-acceptable polymorphism

PhyloP values vary between -14 and +6, the closer the value is to +6, the more probable that the nucleotide is conserved

PhastCons values vary between 0 and 1, the closer the value is to 1, the more probable that the nucleotide is conserved

The effect of splice site mutation was assessed using two tools. Using the Human Splicing Finder (http://www.umd.be/HSF/), the consensus values for G (wild-type) and A (mutant) nucleotides at nt 1084 were calculated as 84.46 (TT**G**gtatgt) and 73.89 (TT**A**gtatgt), respectively, where values of >80 are considered as indicating strong splice site, those of 70–80 as less strong splice site and of 65–70 as weak splice sites. Further, using this tool, reductions of 10% or larger for a genomic variation in any position, or reduction of 7% for a variation in any position +4 are considered as likely to affect splicing [19]. The other tool, MutPred Splice (http://mutdb.org/mutpredsplice/about.htm), gives a score, whose values ≥0.6 predict variants which disrupt splicing [20]. The splice site change observed in our study gave a score of 0.88 and was predicted to lead to loss of natural 5'-splice site with a statistical P value of <0.000001. Thus both the bioinformatics tools used to assess the significance of the observed splice-site mutation suggested that this was likely to alter the splicing of messenger RNA of the *UGT1A1* gene.

### Characterization of mutation at splice site

**Fig 4:**
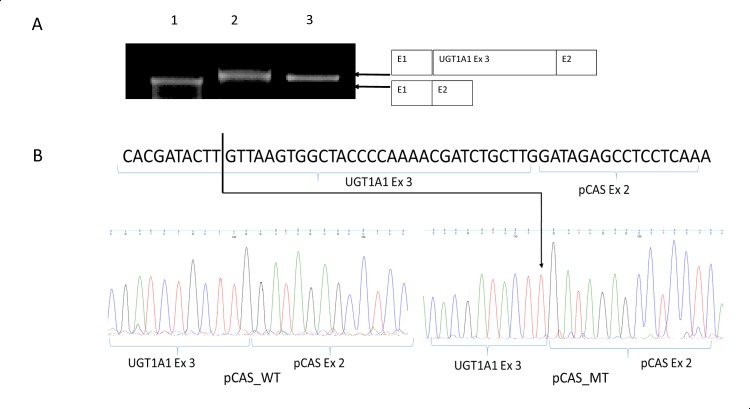
Molecular characterization of c.1084G>A mutation. (A) Results of RT-PCR performed on RNA isolated from transfected COS7 cells. Lane 1: empty pCAS2 vector, lane 2: pCAS_WT vector, and lane 3: pCAS_MT vector. (B) Sequence chromatograms of products of RT-PCR from COS7 transfectants.

The only subject with UH who was homozygous for c.1084G>A (p.Gly362Ser) mutation was subjected to *in vitro* characterization of this mutation to determine whether it caused a defect in mRNA splicing, using pCAS2 minigene. Results indicated that the mutation results in only a minor difference in the size of mature transcript as compared to the control DNA sequence (Fig 4A). Sequencing of specific bands of RT-PCR from pCAS_WT and pCAS_MT revealed a 31-bp deletion from 3’-end of exon 3 in pCAS_MT, whereas pCAS_WT was efficiently spliced (Fig 4B). This result confirms that c.1084G>A mutation leads to abolition of the natural splice site, and the splicing machinery instead chooses another cryptic splice site at 1053 position. This leads to splicing 31 bp before the natural splice site, and causes a frameshift deletion. This mutation leads to p.Lys353Thr and forms a stop codon after three amino acids (p.Lys353ThrfsX3).

## Discussion

In our study, of the 71 subjects with non-hemolytic UH, 65 were homozygous for the well-known A(TA)_7_TAA variant allele in the *UGT1A1* promoter region, a change that is by itself sufficient to cause the condition. Eleven of these 65 subjects also showed additional exonic variations, including 10 who had variations that have been previously reported but are mostly infrequent. Of the remaining 6 subjects with UH who either lacked or had only one copy of the A(TA)_7_TAA variant promoter allele, findings that could not explain the UH phenotype, four showed exonic variations, including two who had a variation that was located at a splice site and was shown in our functional assay to alter splicing.

The variant A(TA)_7_TAA allele in the promoter region of *UGT1A1* gene has been shown to be associated with reduced expression of this gene leading to reduced bilirubin glucuronidation activity [4]. This allele in homozygous form is the most common genetic change associated with the UH phenotype around the world. In Africa, an even more extreme allele with eight TA repeats has been identified and is associated with even lower bilirubin glucuronidation activity [23]. In our study, more than 90% of study subjects with UH of GS variety had the homozygous (TA)_7_ promoter genotype. This is consistent with data from other parts of the world. We, however, did not find the (TA)_8_ promoter variant, which was found to have an allelic frequency of 6% in a group of 77 infants with neonatal hyperbilirubinemia in a previous Indian study [7]; however, other previous Indian studies too did not find this allele [6, 24, 25].

Our findings of most interest are those from six subjects with UH who did not have the variant A(TA)_7_TAA promoter in homozygous form. Since UH in these patients could not be explained by genetic changes in the promoter region, other variations in these patients may be suspected as being responsible for the UH phenotype. In fact, four of six such patients did have variations in the exonic regions or splice sites.

In this context, our most important finding was the presence of a c.1084G>A change in two subjects with UH–in homozygous form in one who lacked the A(TA)_7_TAA *UGT1A1* promoter allele altogether, and in heterozygous form in another who had only one copy of the variant promoter allele. This strongly suggests that the c.1084G>A change is pathogenic, and may have contributed to the occurrence of hyperbilirubinemia in these two subjects. This mutation would lead not only to a change from glycine to serine at amino acid location 362, but could also influence splicing of the messenger RNA for the *UGT1A1* gene.

The ultimate proof that this mutation was pathogenic would require data on expression of the *UGT1A1* mRNA, sequencing of the mRNA to prove altered splicing, and the amount and activity of the UGT1A1 protein. Unfortunately, however, *UGT1A1* gene is expressed mainly in the liver, and hence such studies would need liver biopsy, an invasive procedure which cannot be justified in patients with UH. In such situations, bioinformatics tools have been shown to be useful to predict whether the observed changes are likely to be of clinical consequence or not. These tools found that both the effects of the observed nucleotide change, i.e. change in primary structure of the protein and the likely effect on splicing, were likely to be deleterious (Table 3), indicating that this mutation is highly likely to be pathogenic.

Functional characterization of c.1084G>A splice-site mutation using pCAS2 splicing minigene reporter assay indicates that natural splice site at 1084 position is abolished and a cryptic splice site at 1053 position is taken up for splicing. This leads to frameshift deletion of 31 nucleotides from the 3’-end of exon 3, causing an amino acid change (p.Lys353Thr) and forming a premature stop codon after 3 amino acids. The N-terminal region of the UGT1A1 protein codes for substrate acceptor site, and 246 amino acids at the C-terminal end encode for interaction site with donor substrate, UDP-glucuronic acid, which is common to all UGT1A proteins [26]. The c.1084G>A mutation in exon 3 is a part of the interaction site for donor substrate; hence, this frameshift deletion, which would lead to truncation in this part of the protein is likely to lead to impair its interaction with UDP glucoronic acid. This could explain the higher levels of serum bilirubin in the subject with this c.1084G>A mutation in homozygous state as compared to those with only promotor variation.

Mutation p.Gly362Ser has been reported previously in a child with CNS2 from India [27]. In that subject, p.Gly362Ser was co-inherited with beta-thalassemia. The fairly high bilirubin level in that child may have been a consequence of the coexistent hemolysis. Based on our observations, we postulate that this mutation leads to only a severe form of GS or a mild form of CNS2 depending on the definition used.

Of the other four other subjects with UH who had heterozygous variant promoter genotype, two had exonic variations, namely p.Arg108Cys and p.Phe504Tyr. Data on the clinical consequences of these variations are scanty. These genetic changes were predicted to have a deleterious effect for the protein activity by five and three, respectively, of the eight bioinformatics prediction softwares used. The finding of these variations is interesting. However, the final assessment of phenotypic significance of these mutations will need to await further functional studies on the variant proteins. The remaining two patients with heterozygous variant promoter genotype lacked any detectable exonic change, and thus the reason for UH in them remains unclear.

Eleven of our 65 subjects with UH and homozygous variant promoter region had other exonic variations, all of which were missense. The most common of these was the p.Gly71Arg variation which was present in 5 subjects in heterozygous form. This variation has previously been identified frequently in subjects with UH in Asia, and particularly in Japan [28]. It is believed to be pathogenic, and has been associated with both neonatal hyperbilirubinemia [29] and UH in adults [30] when present in homozygous state or in compound heterozygous form with the variant promoter allele. The presence of this variation in subjects with homozygous variant promoter genotype may reduce the activity of UGT1A1 enzyme further, thus increasing the probability of UH [8]. All our five subjects with this variation were homozygous for the variant promoter allele, making it difficult to ascribe a clear pathogenic role to this variation.

Two other subjects with UH and homozygous variant promoter region had a p.Val225Gly mutation, which has been previously described but whose pathogenic status remains unclear [31]. In four other such subjects, we found missense exonic variations (p.Arg108Cys, p.Ile159Thr, p.Thr205Asn and p.Glu463Val). These observations add to the available information on polymorphisms in the *UGT1A1* gene around the world, and particularly in the Indian population. Our findings however do not permit determination of pathogenic significance of these variations at this stage.

An additional finding of interest from our study may be the absence of A(TA)_8_TAA promoter variant and of a variation at amino acid position 214 in all our 71 subjects. In a previous study from northern India [7], a c.214G>C (p.Ala72Pro) polymorphism was reported in all the 50 neonates, including 29 healthy and 21 with hyperbilirubinemia, in whom this region was sequenced. However, this change has not been reported in any other study either from India or from another part of the world, suggesting that the previous observation may have been an artefact.

In conclusion, our study revealed several variations in Indian subjects with GS, including three (p.Arg108Cys, p.Gly362Ser and p.Phe504Tyr) which were found in subjects in whom promoter region genotype was inadequate to explain the UH phenotype. These observations add to the already available information on genetic variations in the *UGT1A1* gene and their association with UH phenotype.
